# Effect of COPD on the Hospital Outcomes and Mortality among Hemorrhagic Stroke Patients. Sex Differences in a Population-Based Study

**DOI:** 10.3390/jcm10112491

**Published:** 2021-06-04

**Authors:** Javier de Miguel-Diez, Marta Lopez-Herranz, Rodrigo Jiménez-García, Valentín Hernández-Barrera, Isabel Jimenez-Trujillo, Jose M. de Miguel-Yanes, Ana Lopez de Andres

**Affiliations:** 1 Respiratory Care Department, Hospital General Universitario Gregorio Marañón, Instituto de Investigación Sanitaria Gregorio Marañón (IiSGM), Universidad Complutense de Madrid, 28040 Madrid, Spain; javier.miguel@salud.madrid.org; 2 Physiotherapy and Podology Nursing Department, Faculty of Nursing, Universidad Complutense de Madrid, 28040 Madrid, Spain; 3 Department of Public Health & Maternal and Child Health, Faculty of Medicine, Universidad Complutense de Madrid, 28040 Madrid, Spain; rodrijim@ucm.es (R.J.-G.); anailo04@ucm.es (A.L.d.A.); 4 Preventive Medicine, Public Health Teaching and Research Unit, Health Sciences Faculty, Universidad Rey Juan Carlos, Alcorcón, 28922 Madrid, Spain; valentin.hernandez@urjc.es (V.H.-B.); isabel.jimenez@urjc.es (I.J.-T.); 5 Internal Medicine Department, Hospital General Universitario Gregorio Marañón, Instituto de Investigación Sanitaria Gregorio Marañón (IiSGM), Universidad Complutense de Madrid, 28040 Madrid, Spain; josemaria.demiguel@salud.madrid.org

**Keywords:** hemorrhagic stroke, COPD, sex-differences, incidence, in-hospital mortality

## Abstract

(1) Background: It is not well known whether there is an association between COPD and hemorrhagic stroke (HS). We aim to analyze the incidence, clinical characteristics, procedures, and outcomes of HS in patients with and without COPD and to assess sex differences. Secondly, to identify factors associated with in-hospital mortality (IHM). (2) Methods: Patients aged ≥40 years hospitalized with HS included in the Spanish National Hospital Discharge Database (2016–2018) were analyzed. Propensity score matching (PSM) was used to compare patients according to sex and COPD status. (3) Results: We included 55,615 patients (44.29% women). Among men with COPD the HS adjusted incidence was higher (IRR 1.31; 95% CI 1.24–1.57) than among non-COPD men. COPD men had higher adjusted incidence of HS than COPD women (IRR 1.87; 95% CI 1.85–1.89). After matching, COPD men had a higher IHM (29.96% vs. 27.46%; *p* = 0.032) than non-COPD men. Decompressive craniectomy was more frequently conducted among COPD men than COPD women (6.74% vs. 4.54%; *p* = 0.014). IHM increased with age and atrial fibrillation, while decompressive craniectomy reduced IHM. (4) Conclusions: COPD men had higher incidence and IHM of HS than men without COPD. COPD men had higher incidence of HS than COPD women. Decompressive craniectomy was more frequently conducted in COPD men than COPD women and this procedure was associated to better survival.

## 1. Introduction

Chronic obstructive pulmonary disease (COPD) is a worldwide prevalent condition that results in a high morbidity, health care costs, and mortality [1,2]. COPD patients suffer concomitant medical comorbidities that increase the risk of hospital admission, result in longer hospital stays and higher mortality in these patients [3]. Most published reports on comorbidities in COPD patients have focused on cardiovascular diseases and cancer, but little on cerebrovascular diseases such as strokes [4].

Strokes are also a major cause of death worldwide and an important source of acquired disability. The modifiable risk factors of stroke include current smoking, alcohol intake, physical inactivity, high waist-to-hip ratio, hypertension, hyperlipidemia, and diabetes mellitus [5].

Several studies have indicated that COPD patients have a higher risk of strokes than the general population [6,7]. The suggested mechanisms for this association are not fully understood. COPD and stroke share modifiable risk factors, such as smoking and both conditions are associated with aging. Furthermore, oxidative stress and systemic inflammation could play a crucial role by promoting cerebral artery dysfunction, vascular insufficiency, and excessive platelet activation consequently increasing the risk of thrombosis [8].

It is not well known whether associations between COPD and strokes are only present for ischemic strokes, the most common subtype, or they are also applicable for HS [9,10]. Using large population-based national data, we might deepen our understanding of the association between COPD and hemorrhagic strokes. In this investigation we aim to: (a) describe and compare, according to the presence of COPD, the incidence, clinical characteristics, use of therapeutic procedures, and in-hospital outcomes of patients with a primary diagnosis of HS admitted to Spanish hospitals in years 2016, 2017, and 2018; (b) analyze sex-differences among COPD patients hospitalized with hemorrhagic stroke; (c) for patients with COPD hospitalized with hemorrhagic stroke, to identify which variables are associated with the risk of dying during the hospitalization. 

## 2. Materials and Methods

### 2.1. Study Design and Data Source

In this retrospective epidemiological observational study, we used the patient’s information included in the hospital discharge reports collected by the Spanish National Hospital Discharge Database (SNHDD). 

The SNHDD is mandatory, so over 95% of public and private hospitals include their discharge reports in this registry. International Classification of Disease version 10 (ICD-10) is used for coding. For each patient, age, sex and a maximum of 20 diagnoses and 20 procedures are collected. More details in the SNHDD are available online [11].

### 2.2. Study Population and Study Variables

Our study population includes all patients aged 40 years or over with a primary diagnosis of HS admitted to the hospital in the period that goes from 1 January 2016 to 31 December 2018 (see ICD-10 codes in Supplementary Table S1).

We stratified the population according to sex and COPD status. We considered a COPD patient all those with an ICD 10 diagnosis code J44.0, J44.1, or J44.9 in any diagnosis position (2–20). 

Age was categorized in four groups (40 to 59 years, 60 to 74 years, and 75 years or over). 

Incidence of hemorrhagic stroke, in hospital mortality (IHM), length of hospital stay (LOHS), and use of decompressive craniectomy were the primary outcome variables.

The calculation of the incidences was carried out as follows. The number of subjects who had been admitted for HS with and without COPD by sex- age groups were obtained from the SNHDD database. The number of subjects in each of these subgroups were divided by the Spanish population with and without COPD in each of these sex- age groups. To calculate the Spanish population with COPD in each of these subgroups, the total population in each subgroup is multiplied by the prevalence of COPD for the corresponding sex- age group. The total number of Spaniards by sex- age group for the period 2016–2018 was obtained from the National Institute of Statistics [12]. The prevalence of COPD by sex- age group has been obtained from the Spanish National Health Survey for the year 2017. [13]. To calculate the incidence in subjects without COPD, the number of cases of HS without COPD was divided by the population without COPD (total population after subtracting the subjects with COPD in each sex- age group).

Comorbidity was measured with the Charlson Comorbidity Index (CCI) using the algorisms described by Sundararajan et al. for administrative databases [14]. Furthermore, cardiovascular risk factors (obesity, hypertension, and lipid metabolism disorders) and specific conditions (atrial fibrillation, anemia, alcohol abuse, depression, nosocomial pneumonia, and sepsis) were identified in any diagnosis position and analyzed. Concerning procedures, we studied decompressive craniectomy (see Table S1 for ICD10 codes).

### 2.3. Matching Method

In this investigation we used propensity score matching (PSM) to obtain comparable baseline characteristics for men and women with COPD and those without COPD, and between men and women with COPD. PSM was conducted with multivariable logistic regression including as matching variables age, clinical conditions present on admission, and year of hospitalization [15]. 

### 2.4. Statistical Analysis

We used descriptive statistics reporting frequencies and proportions for categorical variables and means with standard deviations (SD) or median with interquartile range (IQR) for continuous variables.

To compare the incidence between study populations adjusted by age and sex when required we used Poisson regression models and provide incidence rate ratios (IRR) with 95% confidence intervals (CI). 

In our analysis, to compere independent subgroups, chi-square tests were applied for categorical variables and Student’s *t*-test or Mann-Whitney test for continuous variables. 

Multivariable logistic regression models, using IHM as the dependent variable, were constructed to identify those variables independently associated with the probability of dying during the hospitalization. We constructed models separately for men and women with and without COPD. Finally, as a sensitivity analysis, we analyzed the effect of sex and COPD after adjusting for possible confounders using the entire database. We obtained adjusted odds ratios (OR) with their 95% confidence intervals (CI).

Stata version 14 (Stata, College Station, TX, USA) was the statistical software used for all data analysis. Significance was set at a two-sided *p*-value of <0.05. 

### 2.5. Ethical Aspects

According to the Spanish legislation, as the SNHDD is mandatory, anonymized, and public access, the approval by an ethics committee is waived. Database requests can be made to the following link [16].

## 3. Results

A total of 55,615 patients (55.71% men and 44.29% women) aged 40 years or over were hospitalized with a primary diagnosis of HS in Spain from year 2016 to 2018. COPD was codified in 4326 patients (7.77%). Among men with HS 9.69% had COPD compared with a 5.36% among women, (*p* < 0.001).

### 3.1. Incidence of Patients Admitted to Hospitals with a Diagnosis of HS According to COPD Status

As can be seen in Table 1, the estimated total incidence of HS per 100,000 persons with COPD was 102.09 ([4326/4,237,437] ∗ 100,000), significantly higher (*p* < 0.001) than among those without COPD with a value of 70.39. ([51,289/72,864,043] ∗ 100,000 = 70.39). 

After age and sex Poisson regression the adjusted IRR was 1.15 (95% CI 1.11–1.19), so in Spain subjects with COPD had a 15% higher risk of being hospitalized with HS than subjects without this condition. When we stratified by sex, we found that among men with COPD the HS adjusted incidence was higher (adjusted IRR 1.31; 95% CI 1.24–1.57) than among non-COPD men. However, among women, the incidences did not differ after adjustment (64.04 women with COPD vs. 60.98 women without COPD; adjusted IRR 1.02; 95% CI 0.97–1.09).

Men with COPD had higher adjusted incidence of HS than COPD women (adjusted IRR 1.87; 95% CI 1.85–1.89). 

### 3.2. Clinical Characteristics and Hospital Outcomes for Men and Women Admitted to Hospitals with a Diagnosis of HS According to COPD Status

As can be seen in Table 2, before PSM, we found that in men with COPD prevalence of intracerebral hemorrhage was higher than in those without COPD (59.32% vs. 55.77%; *p* < 0.001) and prevalence of subarachnoid hemorrhage was lower in men with COPD (9.69% vs. 14.17%; *p* < 0.001). Men with COPD were older (75.47; SD = 10.69 years) than men without COPD (70.82; SD = 13.22 years), and men with COPD also had a higher mean CCI (both *p* < 0.001). COPD men had higher prevalence of most concomitant conditions analyzed. The crude IHM was 29.96% for men with COPD and 23.99% for men without COPD (*p* < 0.001).

After PSM (Table S2), the distribution of concomitant conditions became very similar beside COPD status (Table 2). However, decompressive craniectomy (5.89% vs. 7.26%; *p* = 0.033) remained lower among men with COPD. The IHM was significantly higher in men with COPD (29.96% vs. 27.46%; *p* = 0.032) after all the possible confounding variables were controlled using the PSM.

The comparison of women hospitalized with HS according to COPD status before PSM are shown in Table 3. 

We observed that the mean age of men and women, with and without COPD, was very similar and around 74 years. However, as described among men, women with COPD had higher prevalence of all comorbidities and conditions than non-COPD women exception made for dementia (5.45% vs. 7.91%; *p* = 0.001). The mean CCI were 0.84 and 0.62 for women with and without COPD (*p* > 0.001). The crude IHM, LOHS, and use of decompressive craniectomy were similar beside the presence of COPD.

As can be seen in Table S3, after PSM, we found that use of decompressive craniectomy (4%), LOHS (7 days), and IHM (30%) remained not significantly different in both women with and without COPD.

### 3.3. Sex Differences in the Clinical Characteristics and Hospital Outcomes for COPD Patients Admitted to Hospitals with a Diagnosis of Hemorrhagic Stroke

As can been seen in Table 4, when we compare overall COPD men with COPD women with hemorrhagic stroke, we observe that men were older (75.47 ± 10.69 vs. 74.12 ± 12.86; *p* < 0.001), with a higher mean CCI (1.04 ± 0.89 vs. 0.84 ± 0.74) and more frequently had diabetes, renal disease, peripheral vascular disease, acute myocardial infarction, whereas women had more obesity and depression. Furthermore, men had a higher prevalence of alcohol abuse than females (12.55% vs. 2.87%; *p* < 0.001). However, no sex differences were found regarding crude decompressive craniectomy, LOHS, or IHM.

As expected after PSM (Table S4), we found that LOHS (8 days) and IHM (30%) were similar in both men and women with COPD. However, decompressive craniectomy was slightly but significantly more frequently conducted among COPD men than women (6.74% in men and 4.54% in women, *p* = 0.014). 

### 3.4. Multivariable Analysis of Variables Associated with IHM among COPD Men and Women with Hemorrhagic Stroke

As can been seen in Table 5, the risk of dying in the hospital increased with age and atrial fibrillation for both sexes with COPD. However, renal disease, congestive heart failure, depression, nosocomial pneumonia, and sepsis were factors only associated with IHM in men with COPD.

Undergoing decompressive craniectomy reduced the IHM in both sexes (in men: OR 0.39; 95% CI 0.26–0.60); in women: OR 0.44; 95% CI 0.13–0.93).

Finally, using the entire database including men and women with HS and after multivariable adjustment (Table S5), we found that the probability of dying for men who suffered COPD was 14% higher (OR 1.12; 95% CI 1.02–1.26) than for non-COPD men. This sensitivity analysis confirms the results of the PSM.

## 4. Discussion

In the current population-based study, COPD patients had increased incidence of HS compared with individuals without COPD. However, we only found differences between men with and without COPD, but not between women with and without this disease. In addition, we evidenced that COPD men had higher incidence of HS than COPD women. Nevertheless, given the retrospective nature of the study and the methodology applied, conclusive information on an increased incidence of HS in COPD patients cannot be provided.

Although the incidence of stroke in COPD has been evaluated in some studies, only a few had made distinction between stroke subtypes. In this regard, there is prior evidence indicating that among COPD patients’ the risk of suffering an HS may be higher than the ischemic stroke risk [17]. Furthermore, Portegies et al. [18] found that COPD is associated with a higher risk of hemorrhagic stroke. Similar results were obtained by Kim et al. [19]. In the same way, Söderholm et al. [20] found that incidences of intracerebral hemorrhage and subarachnoid hemorrhage were increased in COPD patients, especially within one year after COPD diagnosis. 

The mechanisms underlying COPD and HS are poorly understood. Both smoking and aging are capable of alter the blood–brain barrier [21,22], thus contributing to the increased risk of HS in COPD patients. Reduced pulmonary function by itself is associated with an increased incidence of stroke, including the hemorrhagic subtype, and this association is independent of smoking status [23,24,25,26]. Arterial hypertension is also a major risk factor for hemorrhagic stroke, and accumulation of blood within the brain may cause damage as a consequence of the increased pressure and mechanical injury [27,28]. In addition, use of anticoagulant therapy in COPD patients could help explain also the association between of HS and COPD [19]. However, Portegies et al. demonstrated, in relationship to the higher risk of both HS in subjects with COPD, that adjustment for hypertension or anti-coagulants at baseline did not change the association, suggesting that small vessel disease can be a likely explanation [18]. Nevertheless, the authors themselves acknowledge that, residual confounding too remains a possibility, either due to single measurements versus lifelong exposures or therapy initiation after baseline.

An important novelty of our study is the analysis of sex-differences among COPD patients hospitalized with hemorrhagic stroke. In fact, we evidenced, for the first time, that COPD men had higher incidence of HS than COPD women. Although there are no previous data on COPD population, previous findings have demonstrated that the risk and absolute number of HS events was also significantly greater in men than in women [29].

In relation to men, decompressive craniectomy was lower among the former. Perhaps, partly because of this, the HMI was significantly higher in men with COPD in our study, since surgical decompression has been associated with a decreased chance of death and increased chance of a favorable outcome [30]. As far as women are concerned, the use of decompressive craniectomy or IHM were not significantly different in both groups. 

We found that LOHS and IHM were similar in both men and women with COPD. However, decompressive craniectomy was significantly more frequently conducted among COPD men than women. In the same way, some previous studies have reported that sex-differences exists regarding the prevention and treatment for primary and secondary stroke, being women less aggressively treated when compared with men [31,32].

Studies of risk factors for mortality in HS are limited [33]. The probability of dying for COPD men who suffered this subtype of stroke in our study was 14% higher than for non-COPD men, but no differences were found in the IHM rate according to COPD status for women. Other authors have also shown that COPD is modestly associated with overall HS mortality but increases in a much higher proportion the mortality after intracerebral hemorrhage [34]. However, previously no distinction has been made between men and women with COPD. Several factors may explain the increased mortality risk among COPD patients. They include associated comorbid conditions, increased risk of dysphagia, aspiration, and pneumonia, and gas exchange disturbances (hypoxemia and hypercapnia) presents in these patients [34]. 

We found that IHM increased with age and atrial fibrillation for males and females with COPD. These risk factors have been already identified as major predictors of mortality in HS patients [35]. By contrast, undergoing decompressive craniectomy reduced the IHM in both sexes in the present study. This surgical intervention has shown to be effective in reducing mortality beside the patient age, although its effectiveness for stroke in COPD patients is still not known [36].

The main strengths of our study are the large sample size, representative of a national population, and the use of a standardized methodology, which has remained stable over time. However, it has several limitations. First, this was a retrospective analysis of a set of administrative data, so there may be coding errors. Second, clinical data, such as severity of HS or airway obstruction and medications, were not available for our analysis. In this sense, the lack of information on the use of anti-thrombotic therapy in the included population is a not negligible defect of the study. COPD is frequently associated with venous thromboembolism or atrial fibrillation, which usually require the patient to take anti-coagulant drugs. Additionally, ischemic heart disease is more common in COPD patients, and patients are often prescribed antiplatelet drugs. Third, we cannot capture stroke deaths outside of the hospital, so we cannot know the long-term mortality or the cost of medical care in this setting. Fourth, a small proportion of patients (4.37%; 189/4326) had a code for COPD exacerbation. This number is too small to assess if suffering an exacerbation could negatively affect the hospital outcome after HS. However, the IHM did not differ significantly (*p* = 0.55) between those with (32.80%) and without (30.02%) a COPD exacerbation. Future investigations should evaluate this issue.

## 5. Conclusions

In conclusion, our study showed that men with of HS and COPD had an increased risk of death compared to those without COPD, although no differences were found in women with and without COPD. IHM increased with age and atrial fibrillation, while decompressive craniectomy reduced it in males and females with COPD. Given the importance of these findings, clinicians should be aware of the effect of COPD in patients with hemorrhagic stroke, with a view to improving their evolution.

## Figures and Tables

**Table 1:** Incidence of HS according to presence of COPD, sex and age groups.

		COPD	No COPD	
Sex	Age Groups	N (Inc/10^5^)	N (Inc/10^5^)	*p*-Value
Men	40–59 years	279 (47.59)	6280 (29.83)	<0.001
	60–74 years	917 (108.24)	9062 (96.73)	0.001
	≥75 years	1808 (244.48)	12,641 (299.19)	<0.001
	All age groups	3004 (138.24)	27983 (80.76)	<0.001
Women	40–59 years	203 (32.59)	4360 (20.48)	<0.001
	60–74 years	384 (53.87)	5656 (55.66)	0.535
	≥75 years	735 (100.85)	13,290 (196.28)	<0.001
	All age groups	1322 (64.04)	23306 (60.98)	0.084
Total	40–59 years	482 (39.86)	10,640 (25.13)	<0.001
	60–74 years	1301 (83.4)	14,718 (75.36)	<0.001
	≥75 years	2543 (173.19)	25,931 (235.82)	<0.001
	All age groups	4326 (102.09)	51,289 (70.39)	<0.001

COPD: Chronic Obstructive Pulmonary Disease. Inc/10^5^: Incidence per 100,000 people with or without COPD.

**Table 2:** Clinical characteristics, use of therapeutic procedures and hospital outcomes before propensity score matching in men patients with hemorrhagic stroke.

Variables	BEFORE PSM		
	COPD	No COPD	*p*-Value
Nontraumatic subarachnoid hemorrhage, *n* (%)	291 (9.69)	3966(14.17)	<0.001
Nontraumatic intracerebral hemorrhage, *n* (%)	1782 (59.32)	15,606 (55.77)	<0.001
Other and unspecified nontraumatic intracranial hemorrhage, *n* (%)	931 (30.99)	8411 (30.06)	0.289
Age, mean (SD)	75.47 (10.69)	70.82 (13.22)	<0.001
CCI, mean (SD)	1.04 (0.89)	0.74 (0.67)	<0.001
Obesity, *n* (%)	218 (7.26)	1384 (4.95)	<0.001
Hypertension, *n* (%)	1700 (56.59)	15,206 (54.34)	0.019
Lipid metabolism disorders, *n* (%)	1105 (36.78)	8302 (29.67)	<0.001
Diabetes, *n* (%)	880 (29.29)	6558 (23.44)	<0.001
Renal disease, *n* (%)	399 (13.28)	2195 (7.84)	<0.001
Atrial fibrillation, *n* (%)	813 (27.06)	4895 (17.49)	<0.001
Congestive heart failure, *n* (%)	250 (8.32)	975 (3.48)	<0.001
Peripheral vascular disease, *n* (%)	229 (7.62)	998 (3.57)	<0.001
Acute myocardial infarction, *n* (%)	126 (4.19)	955 (3.41)	0.027
Dementia, *n* (%)	155 (5.16)	1235 (4.41)	0.060
Anemia, *n* (%)	94 (3.13)	531 (1.9)	<0.001
Alcohol abuse, *n* (%)	377 (12.55)	2534 (9.06)	<0.001
Depression, *n* (%)	141 (4.69)	805 (2.88)	<0.001
Sepsis, *n* (%)	47 (1.56)	407 (1.45)	0.633
Nosocomial pneumonia, *n* (%)	82 (2.73)	602 (2.15)	0.003
Decompressive craniectomy, *n* (%)	177 (5.89)	1789 (6.39)	0.284
LOHS, median (IQR)	8 (12)	7 (12)	0.278
In-hospital mortality, *n* (%)	900 (29.96)	6712 (23.99)	<0.001

PSM: Propensity Score Matching. COPD: Chronic Obstructive Pulmonary Disease. CCI: Charlson comorbidity index; LOHS: length of hospital stay.

**Table 3:** Clinical characteristics, use of therapeutic procedures, and hospital outcomes before propensity score matching in women patients with hemorrhagic stroke.

Variables	BEFORE PSM		
	COPD	No COPD	*p*-Value
Nontraumatic subarachnoid hemorrhage, *n* (%)	305 (23.07)	5688 (24.41)	0.271
Nontraumatic intracerebral hemorrhage, *n* (%)	790 (59.76)	12,680 (54.41)	<0.001
Other and unspecified nontraumatic intracranial hemorrhage, *n* (%)	227 (17.17)	4938 (21.19)	<0.001
Age, mean (SD)	74.12 (12.86)	73.92 (13.63)	0.602
CCI, mean (SD)	0.84 (0.74)	0.62 (0.50)	<0.001
Obesity, *n* (%)	169 (12.78)	1185 (5.08)	<0.001
Hypertension, *n* (%)	753 (56.96)	12,294 (52.75)	0.003
Lipid metabolism disorders, *n* (%)	489 (36.99)	6860 (29.43)	<0.001
Diabetes, *n* (%)	312 (23.6)	4156 (17.83)	<0.001
Renal disease, *n* (%)	139 (10.51)	1460 (6.26)	<0.001
Atrial fibrillation, *n* (%)	340 (25.72)	4300 (18.45)	<0.001
Congestive heart failure, *n* (%)	125 (9.46)	893 (3.83)	<0.001
Peripheral vascular disease, *n* (%)	41 (3.1)	351 (1.51)	<0.001
Acute myocardial infarction, *n* (%)	25 (1.89)	295 (1.27)	0.051
Dementia, *n* (%)	72 (5.45)	1844 (7.91)	0.001
Anemia, *n* (%)	55 (4.16)	661 (2.84)	0.005
Alcohol abuse, *n* (%)	38 (2.87)	358 (1.54)	<0.001
Depression, *n* (%)	147 (11.12)	1761 (7.56)	<0.001
Sepsis, *n* (%)	12 (0.91)	235 (1.01)	0.721
Nosocomial pneumonia, *n* (%)	33 (2.49)	381 (1.63)	0.082
Decompressive craniectomy, *n* (%)	60 (4.54)	1115 (4.78)	0.684
LOHS, median (IQR)	9 (15)	8 (13)	0.148
In-hospital mortality, *n* (%)	404 (30.56)	6861 (29.44)	0.385

PSM: Propensity Score Matching. COPD: Chronic Obstructive Pulmonary Disease. CCI: Charlson comorbidity index; LOHS: length of hospital stay.

**Table 4:** Clinical characteristics, use of therapeutic procedures, and hospital outcomes before propensity score matching among COPD patients with HS according to sex.

Variables	BEFORE PSM		
	Men	Women	*p*-Value
40–59 years, *n* (%)	279 (9.29)	203 (15.36)	<0.001
60–74 years, *n* (%)	917 (30.53)	384 (29.05)	0.328
≥75 years, *n* (%)	1808 (60.19)	735 (55.6)	0.005
Age, mean (SD)	75.47 (10.69)	74.12 (12.86)	<0.001
CCI, mean (SD)	1.04 (0.89)	0.84 (0.74)	<0.001
Obesity, *n* (%)	218 (7.26)	169 (12.78)	<0.001
Hypertension, *n* (%)	1700 (56.59)	753 (56.96)	0.822
Lipid metabolism disorders, *n* (%)	1105 (36.78)	489 (36.99)	0.897
Diabetes, *n* (%)	880 (29.29)	312 (23.6)	<0.001
Renal disease, *n* (%)	399 (13.28)	139 (10.51)	0.011
Atrial fibrillation, *n* (%)	813 (27.06)	340 (25.72)	0.357
Congestive heart failure, *n* (%)	250 (8.32)	125 (9.46)	0.222
Peripheral vascular disease, *n* (%)	229 (7.62)	41 (3.1)	<0.001
Acute myocardial infarction, *n* (%)	126 (4.19)	25 (1.89)	<0.001
Dementia, *n* (%)	155 (5.16)	72 (5.45)	0.697
Anemia, *n* (%)	94 (3.13)	55 (4.16)	0.087
Alcohol abuse, *n* (%)	377 (12.55)	38 (2.87)	<0.001
Depression, *n* (%)	141 (4.69)	147 (11.12)	<0.001
Sepsis, *n* (%)	47 (1.56)	12 (0.91)	0.086
Nosocomial pneumonia, *n* (%)	82 (2.73)	33 (2.49)	0.130
Decompressive craniectomy, *n* (%)	177 (5.89)	60 (4.54)	0.072
LOHS, median (IQR)	8 (12)	9 (15)	0.165
In-hospital mortality, *n* (%)	900 (29.96)	404 (30.56)	0.692

PSM: Propensity Score Matching. COPD: Chronic Obstructive Pulmonary Disease. CCI: Charlson comorbidity index; LOHS: length of hospital stay.

**Table 5:** Multivariable logistic regression analysis of factors associated with in hospital mortality among COPD patients with HS according to sex.

	COPD Men	COPD Women	Both
Variables	OR (95% CI)	OR (95% CI)	OR (95% CI)
40–59 years	1	1	1
60–74 years	1.09 (0.79–1.52)	1.82 (1.19–2.79)	1.33 (0.98–1.81)
≥75 years	1.6 (1.17–2.19)	2.59 (1.75–3.83)	1.94 (1.46–2.57)
Renal disease	1.28 (1.02–1.6)	-	-
Atrial fibrillation	1.41 (1.18–1.69)	1.22 (1.01–1.50)	1.27 (1.05–1.53)
Congestive heart failure	1.35 (1.00–1.81)	-	-
Depression	1.33 (0.92–1.92)	-	-
Sepsis	3.68 (2–6.79)	-	-
Nosocomial pneumonia	1.38 (1.18–1.75)	-	1.16 (1.02–1.29)
Decompressive craniectomy	0.39 (0.26–0.6)	0.44 (0.30–0.93)	0.41 (0.32–0.91)

NA: Not available. Only variables with significant results in the multivariable regression are shown in the table.

## Data Availability

At the link https://www.mscbs.gob.es/estadEstudios/estadisticas/estadisticas/estMinisterio/SolicitudCMBDdocs/2018_Formulario_Peticion_Datos_RAE_CMBD.pdf (accessed on 12 April 2021). Any investigators can make a justified request to the Spanish Ministry of Health for the databases of the SNHDD.

## Supplementary Materials

The following are available online at https://www.mdpi.com/article/10.3390/jcm10112491/s1, Table S1: International Classification of Disease 10th edition (ICD-10) codes for the clinical diagnosis and procedures used in this investigation. Table S2. Clinical characteristics, use of therapeutic procedures and hospital outcomes after propensity score matching in men patients with hemorrhagic stroke. Table S3. Clinical characteristics, use of therapeutic procedures and hospital outcomes after propensity score matching in women patients with hemorrhagic stroke. Table S4. Clinical characteristics, use of therapeutic procedures and hospital outcomes after propensity score matching among COPD patients with HS according to sex. Table S5. Logistic regression factors associated with IHM after HS among all patients and according to the presence of COPD to assess the sex differences.
