# Supplementary material for: Effect of COPD on the Hospital Outcomes and Mortality among Hemorrhagic Stroke Patients. Sex Differences in a Population-Based Study
Source: J Clin Med. 2021 Jun 4;10(11):2491. doi: 10.3390/jcm10112491 (PMC8200115; doi:10.3390/jcm10112491)
Supplement: Supplementary file 1 [file jcm-10-02491-s001.zip › jcm-1217299-supplementary.pdf]

Table S1. International Classification of Disease 10th edition (ICD-10) codes for the clinical diagnosis and procedures used in this investigation.

**Clinical diagnosis and procedures**	**ICD-10 codes**
Nontraumatic subarachnoid hemorrhage	I60.0, I60.00; I60.01; I60.02; I60.1; I60.10, I60.11, I60.12, I60.2, I60.3; I60.30, I60.31, I60.32, I60.4, I60.5, I60.50, I60.51, I60.52, I60.6, I60.7, I60.8, I60.9
Nontraumatic intracerebral hemorrhage	I61.0, I61.2, I61.3, I61.4, I61.5, I61.6, I61.8, I61.9
Other and unspecified nontraumatic intracranial hemorrhage	I62.0, I62.00, I62.01, I62.02, I62.03, I62.1, I62.9
Obesity	E66.X
Hypertension	I10, I16.6
Lipid metabolism disorders	E78.0X-E78.5
Atrial fibrillation	I48.0, I48.1, I48.2, I48.91
Anemia	D50.0, D50.8, D50.9, D51.x-D53.x
Alcohol abuse	F10, E52, G62.1, I42.6, K29.2, K70.0, K70.3, K70.9, T51.x, Z50.2, Z71.4, Z72.1
Depression	F20.4, F31.3-F31.5, F32.x, F33.x, F34.1, F41.2, F43.2
Sepsis	R65.20, R65.21, A40.X, A41.XX
Nosocomial pneumonia	J12-J18, J95.851
Mechanical ventilation	5A1945Z, 5A1955Z, 5A1935Z,5A09357, 5A09457, 5A09557
Decompressive craniectomy	00J00ZZ, 00W00JZ, 00W00KZ, 0N800ZZ, 0N803ZZ, 0N804ZZ, 0NC10ZZ, 0NC13ZZ, 0NC14ZZ, 0NC30ZZ, 0NC33ZZ, 0NC34ZZ, 0NC40ZZ, 0NC43ZZ, 0NC44ZZ, 0NC50ZZ, 0NC53ZZ, 0NC54ZZ, 0NC60ZZ, 0NC63ZZ, 0NC64ZZ, 0NC70ZZ, 0NC73ZZ, 0NC74ZZ, 0NH00MZ, 0NH03MZ, 0NH04MZ, 0NP000Z, 0NP004Z, 0NP005Z, 0NP007Z, 0NP007Z, 0NP00KZ, 0NP00SZ, 0NP030Z, 0NP034Z, 0NP037Z, 0NP03KZ, 0NP03SZ, 0NP040Z, 0NP044Z, 0NP047Z, 0NP04KZ, 0NP04SZ, 0NP0X4Z, 0NP0XSZ, 0NW000Z, 0NW004Z, 0NW005Z, 0NW007Z, 0NW00JZ, 0NW00KZ, 0NW00MZ, 0NW00SZ, 0NW030Z, 0NW034Z, 0NW035Z, 0NW037Z, 0NW03JZ, 0NW03KZ, 0NW03MZ, 0NW03SZ, 0NW040Z, 0NW044Z, 0NW045Z, 0NW047Z, 0NW04JZ, 0NW04KZ, 0NW04MZ, 0NW04SZ, 0W9100Z, 0W010ZZ, 0W9130Z, 0W913ZZ, 0W9140Z, 0W914ZZ, 0WC10ZZ, 0WC13ZZ, 0WC14ZZ, 0WH10YZ, 0WH13YZ, 0WH14YZ, 0WJ10ZZ, 0WP100Z, 0WP101Z, 0WP10JZ, 0WP10YZ, 0WP130Z, 0WP131Z, 0WP13JZ, 0WP13YZ, 0WP140Z, 0WP141Z, 0WP14JZ, 0WP14HZ, 0WW00Z, 0WW101Z, 0WW103Z, 0WW10JZ, 0WW10YZ, 0WW130Z, 0WW131Z, 0WW133Z, 0WW13JZ, 0WW13YZ, 0WW140Z, 0WW141Z, 0WW143Z, 0WW14JZ, 0WW14YZ, 0N500ZZ, 0N503ZZ, 0N504ZZ, 0NB00ZZ, 0NB03ZZ, 0NB04ZZ, 0NT10ZZ, 0NT30ZZ, 0NT40ZZ, 0NT50ZZ, 0NT60ZZ, 0NT70ZZ, 009100Z, 00910ZZ, 00C10ZZ, 00C13ZZ, 00C14ZZ, 009000Z, 00900ZZ, 009030Z, 00903ZZ, 009040Z, 00904ZZ, 00C00ZZ, 00C03ZZ, 00C04ZZ, 00H003Z, 00H003Z, 00H00YZ, 00H032Z, 00H033Z, 00H03YZ, 00H042Z, 00H043Z, 00H04YZ, 00H602Z, 00H603Z, 00H60YZ, 00H632Z, 00H633Z, 00H63YZ, 00H642Z, 00H643Z, 00H64YZ, 00P000Z, 00P002Z, 00P003Z, 00P007Z, 00P00JZ, 00P00KZ, 00P00YZ, 00P030Z, 00P032Z, 00P033Z, 00P037Z, 00P03JZ, 00P03KZ, 00P03YZ, 00P040Z, 00P042Z, 00P043Z, 00P047Z, 00P04JZ, 00P04KZ, 00P04YZ, 00P600Z, 00P602Z, 00P603Z, 00P60YZ, 00P630Z, 00P632Z, 00P633Z, 00P63YZ, 00P640Z, 00P642Z, 00P643Z, 00P64YZ, 00P6X2Z, 00W000Z, 00W002Z, 00W003Z, 00W007Z, 00W00MZ, 00W00YZ, 00W030Z, 00W032Z, 00W033Z, 00W037Z, 00W03JZ, 00W03KZ, 00W03MZ, 00W03YZ, 00W040Z, 00W042Z, 00W043Z, 00W047Z, 00W04JZ, 00W04KZ, 00W04MZ, 00W04YZ, 00W600Z, 00W602Z, 00W603Z, 00W60MZ, 00W60YZ, 00W630Z, 00W632Z, 00W633Z, 00W63MZ, 00W63YZ, 00W640Z, 00W642Z, 00W643Z, 00W64MZ, 00W64YZ, 00B70ZZ, 00B73ZZ, 00B74ZZ, 0500ZZ, 00503ZZ, 00504ZZ, 00B00ZZ, 00B03ZZ, 00B04ZZ.

Table S2. Clinical characteristics, use of therapeutic procedures and hospital outcomes after propensity score matching in men patients with hemorrhagic stroke.

Variables	AFTER PSM		
	**COPD**	**No COPD**	**p-value**
Nontraumatic subarachnoid hemorrhage, n(%)	291(9.69)	282(9.39)	0.752
Nontraumatic intracerebral hemorrhage, n(%)	1782(59.32)	1794(59.72)	0.932
Other and unspecified nontraumatic intracranial hemorrhage, n(%)	931(30.99)	928(30.89)	0.911
Age, mean (SD)	75.47(10.69)	75.45(10.92)	0.945
CCI, mean (SD)	1.04(0.89)	0.99(0.87)	0.061
Obesity, n(%)	218(7.26)	202(6.72)	0.418
Hypertension, n(%)	1700(56.59)	1679(55.89)	0.585
Lipid metabolism disorders, n(%)	1105(36.78)	1096(36.48)	0.810
Diabetes, n(%)	880(29.29)	881(29.33)	0.977
Renal disease, n(%)	399(13.28)	411(13.68)	0.650
Atrial fibrillation, n(%)	813(27.06)	821(27.33)	0.817
Congestive heart failure, n(%)	250(8.32)	249(8.29)	0.963
Peripheral vascular disease, n(%)	229(7.62)	225(7.49)	0.845
Acute myocardial infarction, n(%)	126(4.19)	124(4.13)	0.897
Dementia, n(%)	155(5.16)	164(5.46)	0.605
Anemia, n(%)	94(3.13)	97(3.23)	0.825
Alcohol abuse, n(%)	377(12.55)	363(12.08)	0.583
Depression, n(%)	141(4.69)	130(4.33)	0.494
Sepsis, n(%)	47(1.56)	49(1.63)	0.837
Nosocomial pneumonia, n(%)	82(2.73)	62(2.06)	0.094
Decompressive craniectomy, n(%)	177(5.89)	218(7.26)	0.033
LOHS, median (IQR)	8(12)	7(11)	0.857
In-hospital mortality, n(%)	900(29.96)	825(27.46)	0.032

PSM: Propensity Score Matching. COPD: Chronic Obstructive Pulmonary Disease. CCI: Charlson comorbidity index; LOHS: length of hospital stay.

Table S3. Clinical characteristics, use of therapeutic procedures and hospital outcomes after propensity score matching in women patients with hemorrhagic stroke.

**Variables**	**AFTER PSM**		
	**COPD**	**No COPD**	**p-value**
Nontraumatic subarachnoid hemorrhage, n(%)	305(23.07)	306(23.15)	0.692
Nontraumatic intracerebral hemorrhage, n(%)	790(59.76)	780(59)	0.645
Other and unspecified nontraumatic intracranial hemorrhage, n(%)	227(17.17)	236(17.85)	0.887
Age, mean (SD)	74.12(12.86)	74.56(12.94)	0.375
CCI, mean (SD)	0.84(0.74)	0.79(0.67)	0.169
Obesity, n(%)	169(12.78)	166(12.56)	0.861
Hypertension, n(%)	753(56.96)	768(58.09)	0.555
Lipid metabolism disorders, n(%)	489(36.99)	502(37.97)	0.601
Diabetes, n(%)	312(23.6)	318(24.05)	0.784
Renal disease, n(%)	139(10.51)	141(10.67)	0.899
Atrial fibrillation, n(%)	340(25.72)	350(26.48)	0.658
Congestive heart failure, n(%)	125(9.46)	124(9.38)	0.947
Peripheral vascular disease, n(%)	41(3.1)	40(3.03)	0.910
Acute myocardial infarction, n(%)	25(1.89)	19(1.44)	0.362
Dementia, n(%)	72(5.45)	67(5.07)	0.663
Anemia, n(%)	55(4.16)	54(4.08)	0.922
Alcohol abuse, n(%)	38(2.87)	36(2.72)	0.814
Depression, n(%)	147(11.12)	140(10.59)	0.662
Sepsis, n(%)	12(0.91)	8(0.61)	0.369
Nosocomial pneumonia, n(%)	33(2.49)	21(1.59)	0.271
Decompressive craniectomy, n(%)	60(4.54)	57(4.31)	0.777
LOHS, median (IQR)	9(15)	8(12)	0.137
In-hospital mortality, n(%)	404(30.56)	422(31.92)	0.450

PSM: Propensity Score Matching. COPD: Chronic Obstructive Pulmonary Disease. CCI: Charlson comorbidity index; LOHS: length of hospital stay

Table S4. Clinical characteristics, use of therapeutic procedures and hospital outcomes after propensity score matching among COPD patients with hemorrhagic stroke according to sex.

Variables	AFTER PSM		
	**Men**	**Women**	**p-value**
40-59 years, n(%)	162(12.25)	203(15.36)	0.021
60-74 years, n(%)	394(29.8)	384(29.05)	0.670
≥75 years, n(%)	766(57.94)	735(55.6)	0.224
Age, mean (SD)	74.44(11.54)	74.12(12.86)	0.500
CCI, mean (SD)	0.86(0.78)	0.84(0.74)	0.550
Obesity, n(%)	150(11.35)	169(12.78)	0.257
Hypertension, n(%)	760(57.49)	753(56.96)	0.783
Lipid metabolism disorders, n(%)	497(37.59)	489(36.99)	0.748
Diabetes, n(%)	327(24.74)	312(23.6)	0.496
Renal disease, n(%)	142(10.74)	139(10.51)	0.850
Atrial fibrillation, n(%)	375(28.37)	340(25.72)	0.125
Congestive heart failure, n(%)	109(8.25)	125(9.46)	0.273
Peripheral vascular disease, n(%)	35(2.65)	41(3.1)	0.485
Acute myocardial infarction, n(%)	23(1.74)	25(1.89)	0.771
Dementia, n(%)	77(5.82)	72(5.45)	0.673
Anemia, n(%)	52(3.93)	55(4.16)	0.767
Alcohol abuse, n(%)	42(3.18)	38(2.87)	0.650
Depression, n(%)	113(8.55)	147(11.12)	0.026
Sepsis, n(%)	10(0.76)	12(0.91)	0.669
Nosocomial pneumonia, n(%)	43(3.26)	33(2.49)	0.018
Decompressive craniectomy, n(%)	89(6.74)	60(4.54)	0.014
LOHS, median (IQR)	8(13)	9(15)	0.154
In-hospital mortality, n(%)	404(30.56)	404(30.56)	0.999

PSM: Propensity Score Matching. COPD: Chronic Obstructive Pulmonary Disease. CCI: Charlson comorbidity index; LOHS: length of hospital stay.

Table S5. Logistic regression factors associated with IHM after hemorrhagic stroke among all patients and according to the presence of COPD to assess the sex differences.

	**Male**	**Female**
**Variables**	**OR (95%CI)**	**OR (95%CI)**
40-59 years	1	1
60-74 years	1(0.8-1.27)	1.49(1.11-1.99)
≥75 years	1.52(1.22-1.89)	2.1(1.61-2.74)
Renal disease	1.28(1.09-1.5)	
Atrial fibrillation	1.45(1.28-1.65)	-
Congestive heart failure	1.41(1.16-1.72)	1.38(1.13-2.07)
Dementia	-	1.58(1.1-2.27)
Peripheral vascular disease	1.23(1-1.52)	-
Sepsis	3.75(2.45-5.72)	-
Decompressive craniectomy	0.36(0.27-0.49)	0.58(0.36-0.93)
COPD	1.12(1.01-1.26)	-

COPD: Chronic Obstructive Pulmonary Disease. NA: Not available Only variables with significant results in the multivariable regression are shown in the table.
